# Prediction Pressure Ulcers in High Care Unit Patients: Evaluating Risk Factors and Predictive Scale Using a Prospective Cross-Sectional Study

**DOI:** 10.1055/s-0043-1777420

**Published:** 2024-02-27

**Authors:** Anies Dewi Wirati Indraswari, Umi Aisyiyah, Kurniawan Kurniawan, Meircurius Dwi Condro Surboyo

**Affiliations:** 1 Intensive Care Unit, Fatmawati Hospital, Cilandak – South Jakarta, Indonesia; 2 Committee of Nursing, Fatmawati Hospital, Cilandak - South Jakarta, Indonesia; 3 Department of Oral Medicine, Faculty of Dental Medicine, Universitas Airlangga, Indonesia

**Keywords:** pressure ulcer, high care unit, prediction, risk factor, Norton, Jackson/Cubbin

## Abstract

**Background** The incidence of ulcer pressure in the high care unit (HCU) was relatively high and could be reliably predicted using tools such as the Norton and Jackson/Cubbin scales. However, other risk factors, such as age, gender, consciousness, systemic condition, duration of treatment, and use of restraint, may contribute to the occurrence of ulcer pressure. This study was conducted to analyze the relationship of various risk factors for pressure ulcers and prediction of ulcer pressure, using Norton and Jackson/Cubbin scale, to incident pressure ulcers in HCU patient.

**Methods** This study utilized a prospective cross-sectional study design to analyze various risk factors for ulcer pressure development in a patient admitted to the HCU, including age, gender, blood profile, consciousness, duration of treatment, and use of restraint. The Norton and Jackson/Cubbin scale was employed to predict pressure ulcers. The relationship between the risk factors and the prediction of pressure ulcer incidents was evaluated using multiple logistic binary regression analysis.

**Result** Both the Norton and Jackson/Cubbin scales predicted a lower risk of pressure ulcer development (60.98 and 99.02%, respectively). This prediction is consistent with the low incidence of pressure injuries found, which is only 4.39%. Furthermore, the relationship between the identified risk factor (gender, duration of treatment in HCU and use of restraint) and the prediction and incident of pressure ulcer was not significant ( *p* > 0.05). Thus, it is suggested that these risk factors may not strong predictors of pressure ulcer development.

**Conclusion** This study's result indicated no significant relationship exists between possible identified risk factors and the development of pressure ulcers in HCU patients. However, the Norton and Jackson/Cubbin scales were reliable predictors of pressure ulcer occurrence, with both scales predicting a lower risk of pressure ulcer development.

## Introduction

Pressure injuries, commonly referred to as pressure sores or decubitus wounds, pose a significant challenge in the realm of healthcare, particularly in the context of hospitalized patients. 1 2 3 These ailments, if left unattended, can lead to severe consequences, including fatalities. However, through the diligent application of appropriate preventive measures, the incidence of pressure injuries can be significantly mitigated. The multifaceted issue of pressure injuries, specifically focusing on their prevalence in critical care patients within high-care units (HCU). 4 The heightened vulnerability of these patients to pressure injuries can be attributed to factors such as prolonged immobilization, medical treatment procedures, and the use of various medical devices.

The impact of pressure injuries on patients is profound, affecting their physical and psychological well-being. These injuries result in pain, 5 and physiological distress, 5 hindering the natural healing process, 6 and potentially exacerbating the prognosis. Furthermore, pressure injuries also place a substantial burden on healthcare facilities, prolonging patient care and escalating the overall cost of treatment. 7

In light of these challenges, it becomes imperative to employ comprehensive and suitable risk assessment strategies to prevent pressure injuries in hospitalized patients. 8 9 Risk assessment is a fundamental step for healthcare professionals, particularly nurses, as it informs the development and implementation of preventive measures. 10 Various assessment tools, such as the Braden scale, 11 Norton scale, 12 13 and Jackson/Cubbin scale, 14 have been used to evaluate the risk of pressure injuries. However, these tools exhibit differences in predictive accuracy, effectiveness, and value. 15 16

The Norton scale, for instance, stands out as an efficient and critical care-specific risk assessment tool designed to address the unique vulnerabilities of critically ill patients. 13 This scale demonstrates a notable ability to predict pressure ulcer occurrence and has been associated with reducing the prevalence of ulcers to below 10%. 17 Yet, it has limitations when used in isolation. 18 19 On the other hand, studies suggest that the Jackson/Cubbin scale may be the most suitable risk assessment tool for HCU settings, but it faces challenges related to predictive value. 20 Additionally, the intensive care unit (ICU) admission and Jackson/Cubbin scale are independent predictors of ICU mortality. 21

The prediction of pressure ulcer occurrence is not solely reliant on the use of measurement scales. An objective assessment of risk factors is vital for accurate prediction and prevention, especially for high-risk patients. 22 Factors such as age, gender, systemic condition, level of consciousness, treatment duration, and the presence or absence of edema play crucial roles in the overall assessment of risk. Therefore, this study aims to explore the relationship between these risk factors and the prediction of pressure injuries, utilizing the Norton and Jackson/Cubbin scales, in the context of HCU patients.

## Materials and Methods

### Sample

The prospective cross-sectional study was used for this study, from May to July 2021. The population under study included all critically ill patients who received treatment in the HCU of RSUP Fatmawati. The study sample comprised patients who met the following criteria: aged above 18 years old, received treatment in HCU for more than 24 hours, and had no clinical signs of pressure injury at admission to the HCU.

### Risk Factor Assessment

The objective examination involved observing the following parameter: blood laboratory examination (hemoglobin, leucocytes, random blood glucose), level of consciousness by the Glasgow Coma Scale (GCS), duration of treatment, restraint use, and the presence or absence of oedema.

### Pressure Injury Assessment

Two different risk assessments for pressure injury development were performed using the Norton and Jackson/Cubbin scale. The Norton scale evaluated physical status, mental status, activity, mobility and incontinence, with the aspect classified on a scale from 1 (disabled) to 4 (able). The Jackson/Cubbin scale assessed age, body weight, skin condition, mental status, mobility, hemodynamics, respiration, nutrition, incontinence, and hygiene as a potential risk factor for pressure injury development. 23

### Data Analysis

A descriptive analysis was conducted to calculate frequencies and proportions. To investigate the impact of different risk factors and pressure ulcer predictions on the occurrence of pressure ulcers injury, a multiple logistic regression test was performed. Prior to conducting the impact test, a binary logistic regression test was conducted to determine which variables were included in the equation model and to determine their strength. The collected data were coded, validated, and analyzed using IBM SPSS Statistic version 23. A *p* -value of less than 0.05 was considered statistically significant.

## Results

### Patient Demography

The total sample for this study consisted of 204 patients, with a slightly higher proportion of male patients (56.10%). The age distribution of the patients showed a predominance in the age groups of 51 to 60 years (29.27%), 61 to 70 years (24.88%), and 41 to 50 years (20.49%; Table 1 ).

**Table 1:** The patient demographic was included in this study

Data	Number (%)
**Gender**	
Male	115 (56.10)
Female	89 (43.41)
Ages (years)	
10–20	3 (1.46)
21–30	13 (6.34)
31–40	17 (8.29)
41–50	42 (20.49)
51–60	60 (29.27)
61–70	51 (24.88)
71–80	12 (5.85)
81–90	6 (2.93)

### Risk Factor Assessments

The blood examination involved several parameters including hemoglobin and leukocytes and random blood glucose. The majority of patients had hemoglobin values greater than 10 g/dL (60.49%), while the average leukocyte count was greater than 10 × 10 ^3^ /µL (61.95%). In terms of fasting blood sugar levels, 39.03% of patients had values in the ranges of 60 to 120 mg/dL and 40.49% patients had value in the range of 120 to 200 mg/dL ( Table 2 ).

**Table 2:** The risk factor observed in sample study

Parameter	Number (%)
Hemoglobin (g/dL)	
< 8	43 (20.98)
8–10	37 (18.05)
> 10	124 (60.49)
Leukocyte (10 ^3^ /µL)	
< 5	12 (5.85)
5–10	65 (31.71)
> 10	127 (61.95)
Fasting blood glucose (mg/dL)	
< 60	3 (1.46)
60–120	80 (39.02)
120–200	83 (40.49)
> 200	38 (18.54)
GCS	
Somnolence	3 (1.46)
Delirium	13 (6.34%)
Apatis	21 (10.24)
Compose mentis	164 (80.0)
Sopor	3 (1.46)
The presence of edema	
Negative	175 (85.37)
Positive	29 (14.15)
Restraint use	
Yes	25 (12.20)
No	179 (87.32)
Duration of treatment (days)	
< 7	122 (59.51)
7–14	76 (37.07)
15–21	4 (1.95)
> 21	2 (0.98)

Abbreviation: GCS, Glasgow Coma Scale.

The patient's general condition was objectively assessed using a GCS, which was classified into five categories. The majority of patients were in a composed mentis state (80.00%), while 10.24% were in a state of apathy. The presence of risk factors for pressure ulcer development, such as edema and restraint use upon admission to the HCU, was also observed. Only 14.15% of patients presented with edema and 12.20% patients were restrained. The duration of treatment in the HCU is also determining factor for pressure ulcer occurrence, The majority of patients were treated for less than 7 days (59.51%), with 37.07% undergoing treatment for 7 to 14 days ( Table 2 ).

### Pressure Injury Assessment and Ulcer Pressure Incident

Using the Norton Scale to make predictions, it was found that all patients in this study were susceptible to developing pressure ulcers. Specifically, 34.15% of patients were categorized as having a low risk, 60.98% were categorized as having a moderate risk, and 4.39% were categorized as having a high risk of developing pressure ulcers. Similarly, prediction using the Jackson-Cubbin scale showed that most patients were at low risk (99.02%) and only 0.49% had no risk of developing pressure ulcers ( Table 3 ).

**Table 3:** The risk assessment of ulcer pressure development

	Number of patient (%)
Norton scale	
> 18 (high risk)	0
14-18 (moderate risk)	70 (34.15)
10-13 (low risk)	125 (60.98)
< 10 (no risk)	9 (4.39)
Jackson-Cubbin scale	
> 37 (high risk)	1 (0.49)
15-37 (moderate risk)	203 (99.02)
< 15 (low risk)	0
Ulcer pressure incident	
Negative	195 (95.61)
Positive	9 (4.39)

During clinical examination, only a small percentage (4.39%) of the 204 observed patients had developed pressure ulcers, while the majority (95.61%) of patients did not exhibit any sign of pressure ulcers ( Table 3 ).

### The Relationship of Risk Factors to the Incidence of Pressure Ulcers

Each variable including age, gender, blood profile, consciousness, duration of treatment, use of restraint, and measurements using the Norton scale and Jackson/Cubbin scale was analyzed. The variables that meet the criteria for inclusion in the multiple logistic regression equation were gender, duration of treatment, and the use of restraints, as they had a *p* -value of less than 0.25 ( Table 4 ).

**Table 4:** The risk factor assessment using binary logistic regression

Variable	*p* -Value
Gender	0.169 a
Age	0.494
Hemoglobin count	0.810
Leucocytes count	0.602
Fasting blood glucose	0.770
Consciousness	0.734
Duration of treatment	0.061 a
The present of edema	0.915
Restrain use	0.110 a
The ulcer pressure injury using Norton scale	0.282
The ulcer pressure injury using Jackson/Cubbin scale	0.774

a Significant value using the multiple binary regression with *p* < 0.25.

Furthermore, to determine the extent of the influence of the risk factor of gender, duration of treatment and restraints on the occurrence of pressure ulcers, a multiple logistic regression test was conducted and the result is presented in Table 5 . The results indicated that none of these risk factors had a significant effect on the occurrence of pressure ulcers in HCU patients, as the *p* -value for each risk factor was greater than 0.05.

**Table 5:** The risk factor assessment using multiple logistic regression

Variable	*p* -Value a
Gender	0.280
Duration of treatment	0.181
Restrain use	0.329

a Significant value using the multiple logistic regression with *p* < 0.05.

## Discussion

The assessment of pressure ulcer risk in hospital settings is a crucial aspect of patient care, particularly in the case of critically ill patients. The Norton scale and the Jackson/Cubbin scale are widely used tools for assessing pressure ulcer risk. 24 25 These scales incorporate multiple risk factors that have been shown to possess high levels of validity and reliability, enabling accurate predictions of pressure ulcer occurrence. 26 27 28 29 30 Additionally, considering internal patient factors, such as underlying disease and nutritional status, has been shown to enhance the predictive ability of these scales. 19 31

Beyond predicting pressure ulcer occurrence, the Norton scale is widely adapted for selecting and predicting treatment success in various conditions, such as implantable cardioverter defibrillators, 32 transcatheter aortic valve implantation in the elderly, 18 and reducing complications in hip arthroplasty in the elderly patients. 33 Furthermore, the Norton scale can even be utilized to determine the duration of hospital stays, particularly in the elderly. 34 However, it should be noted that a report has shown that the predictive power of these scales becomes invalid if the duration of hospitalization or treatment in HCU exceeds several weeks. Thus, it is important to consider these limitations when utilizing the Norton and Jackson/Cubbin scales for pressure ulcer risk assessment. 17

The patient's condition including age, body weight, physical status, mental status, mobility and incontinence, skin condition, hemodynamics, respiration, nutrition, and hygiene become the main aspects in predicting ulcer pressure in Norton and Jackson/Cubbin tool prediction. 23 Recently, it is also considered that gender, the duration of treatment in HCU, and the systematic underlying condition like anemia, hypoalbuminemia, diabetes, and hypotension also contribute to the occurrence of ulcer pressure. 35 Another study concludes that no single factor contributes to the development of ulcer pressure. 36 The other risk factors, related to use of medical devices, are strongly related to the incident the pressure ulcer. 37

In this study, various risk factors used as predictors on the Norton and Jackson/Cubbin scale provided results that matched the incidence of ulcer pressure rates. Various risk factors were analyzed, and the incidence rate of ulcer pressure was very small, which was only 4.39%. This in inline with prediction by Norton and Jackson/Cubbin, which only predicted ulcer pressure, who have high risk of 0% and 0.49%, respectively. Allegations of association or association with other risk factors did not yield significant results. Of the various risk factors that exist, it seems that age, duration of treatment, and restraints are probable factors in the occurrence of pressure ulcers. Research by Latimer et al resulted in 10.8% of older patients having a pressure injury within the first 36 hours of hospital admission. Age and duration of treatment may contribute to the prevalence of pressure injury among older people within the first 36 hours of hospitalisation. 38

Limited knowledge of other factors such as hematological profile (hemoglobin count, leucocyte count, fasting blood glucose count), the level of consciousness, and the presence or absence of oedema in the skin are not closely related to the occurrence of pressure ulcers. In the animal model, the relationship between hemoglobin level, body weight, and healing ulcer is related to the intake of protein. 39 The hematological profile, like hemoglobin count, leucocyte count, and fasting blood glucose count, that reflected the systemic condition, is influenced by several factors, like age and nutritional diet. 40

Based on the data, it seems that this study may have limitations in terms of sample size. In order to determine the relationship between various risk factors and the development of pressure ulcers, a large-scale sample may be necessary. Additionally, the age, duration of treatment, and restraint factor may be important
